# Mapping riparian zone macro litter abundance using combination of optical and thermal sensor

**DOI:** 10.1038/s41598-022-09974-4

**Published:** 2022-04-12

**Authors:** Fatwa Ramdani, Riswan Septriayadi Sianturi, Muhammad Tanzil Furqon, Mahardeka Tri Ananta

**Affiliations:** 1 Program in Economic and Public Policy, Graduate School of Humanities and Social Sciences, University of Tsukuba, Tsukuba City, 305-8571 Japan; 2 Geoinformatics Research Group, Faculty of Computer Science, Brawijaya University, Malang City, 65145 Indonesia; 3 Media, Game & Mobile Research Group, Faculty of Computer Science, Brawijaya University, Malang City, 6515 Indonesia

**Keywords:** Environmental sciences, Environmental impact

## Abstract

A significant increase in the world's population will lead to an increase in consumption and, therefore, an increase in global waste. Various attempts have been made to monitor and map waste, but the proposed approaches are difficult and complicated, and they incur high costs. In this study, to overcome limitations in monitoring and mapping plastic waste, using combined optical and thermal sensors installed on drones is proposed. The study area is the riparian zone, or the zone around the river, where the accumulation of plastic waste at the mouth of the river eventually reaches the sea. The image data obtained were processed using machine learning methods to produce high accuracy and precision. To determine the effectiveness of the proposed method, an accuracy assessment was conducted. The results of this study indicate that the combination of optical and thermal sensors provides the best accuracy compared to using only single optical or thermal image data.

## Introduction

The world population will increase to more than nine billion by 2050^1^. According to the United Nations (UN), nine countries will contribute to more than half of the population growth between 2019 and 2050, and Indonesia is one such country^1^. Rapid population growth will bring challenges for sustainable development. Consumption will increase along with pollution from anthropogenic sources, such as plastics.

Urban development and consumption are the main factors driving an increase in pollution. The production of anthropogenic mass such as plastic, concrete, bricks, asphalt, and metal have caused human-made materials to exceed the entire living biomass on Earth^2^. Furthermore, human-made plastic mass outweighs that of animals and natural biomass by 8 gigatonnes (Gt) and 4 Gt, respectively. Buildings and infrastructure outweigh trees and shrubs by 1100 Gt and 900 Gt, respectively^2^.

Macro litter is any anthropogenic, manufactured, or processed solid material that is discarded, disposed of, or abandoned, and enters the water environment^3^. This pollutant threatens the environment as well as human health. This study will provide state-of-the-art mapping technology to monitor and quantify macro litter in the riparian zone of the Brantas River in Malang City, East Java, in Indonesia. It is important to maintain a healthy riparian zone because it contains diverse plant and aquatic species, and terrestrial wildlife. The riparian zone also helps to maintain the surface temperature, filter the water, control floods, provide habitat for plants and animals, prevent landslides, and decrease sedimentation^4^.

With a population of more than 800,000 people^5^, Malang City is experiencing rapid development. It is the second most populous city in the East Java Province after Surabaya City and the third-largest city in the economy after Surabaya and Kediri. The Brantas River is a large main river flowing through Malang City. This river provides substantial resources for the city’s inhabitants, such as living space and socio-economic activities. According to a report by the Malang City Environmental Services, the river has been polluted mostly by plastic waste^6^. Furthermore, there are more than 25 locations on the Malang River that are polluted^7^, and most of the plastic waste that is dumped into the river and accumulates will have a negative impact. Decreasing surface water quality, increasing flood risk, vegetation loss, increasing surface temperature, and declining biodiversity affect hundreds of plant and aquatic species, and the terrestrial wildlife that depends on the river^4^.

Currently, there is no monitoring system available to quantify and map the spatial and temporal distribution of macro litter abundance in the Brantas River riparian zone. Existing methods of monitoring and mapping the riparian zone have been published^8–14^, however, research regarding monitoring and mapping macro litter abundance is limited.

Space-based imagery for monitoring and detecting floating plastic litter on the sea surface area^15–17^ and marine plastic debris^18, 19^ has been proposed, and unmanned aerial systems (UAS) have been used on a sandy beach^20^ and coastal zone^21^. These studies were conducted in marine and coastal environments for large size plastic debris.

Themistocleous et al.^15^ used a Sentinel-2 image with a spatial resolution of 10 m to detect plastic bottles that were placed in the sea near the Old Port of Limassol in Cyprus, and Gonçalves et al.^20^ used a low-cost Red–Green–Blue (RGB) camera with a spatial resolution of 5.5 mm onboard DJI Phantom 4.

Recently, four key machine learning methods have been proposed by researchers. These were, Extreme Gradient Boosting (XGBoost) that was applied to map the boreal landscape^22^, Support Vector Machine (SVM) that was applied to map oil palm plantations^23^, Random Forest (RF) that was applied to map vegetation types in semi-arid riparian regions^12^, and Artificial Neural Network (ANN)^24^ that was applied to map dense urbanised areas. However, few studies have applied these methods to map and quantify macro litter in riparian zones.

With the knowledge that there is scant research that has monitored macro litter in the riparian zone using a combination of very high-resolution optical and thermal aerial images with cost-effective UAS, here it is hypothesised that the combination of thermal and optical images will produce higher accuracy of macro litter abundance detection than using only a single optical or thermal image.

The main objective of this study is to propose and evaluate a simple and cost-effective geospatial technology approach based on high-resolution aerial images and a cost-effective UAS method for automatically mapping macro litter abundance in the riparian zone. The specific objectives of this study are as follows:

- To evaluate the performance of three commonly used object-oriented machine learning classifiers, namely, Extreme Gradient Boosting (XGBoost), Support Vector Machine (SVM), Random Forest (RF), and Artificial Neural Network (ANN).
- To contribute to advances in remote sensing surveys by optimising automated detection on very high-resolution aerial images (optical and thermal) UAS-derived orthomosaics.
- To assist environmental pollution monitoring programs and contribute to the research and evaluation of mitigation measures.

## Material and methods

### Study area

**Figure 1:**
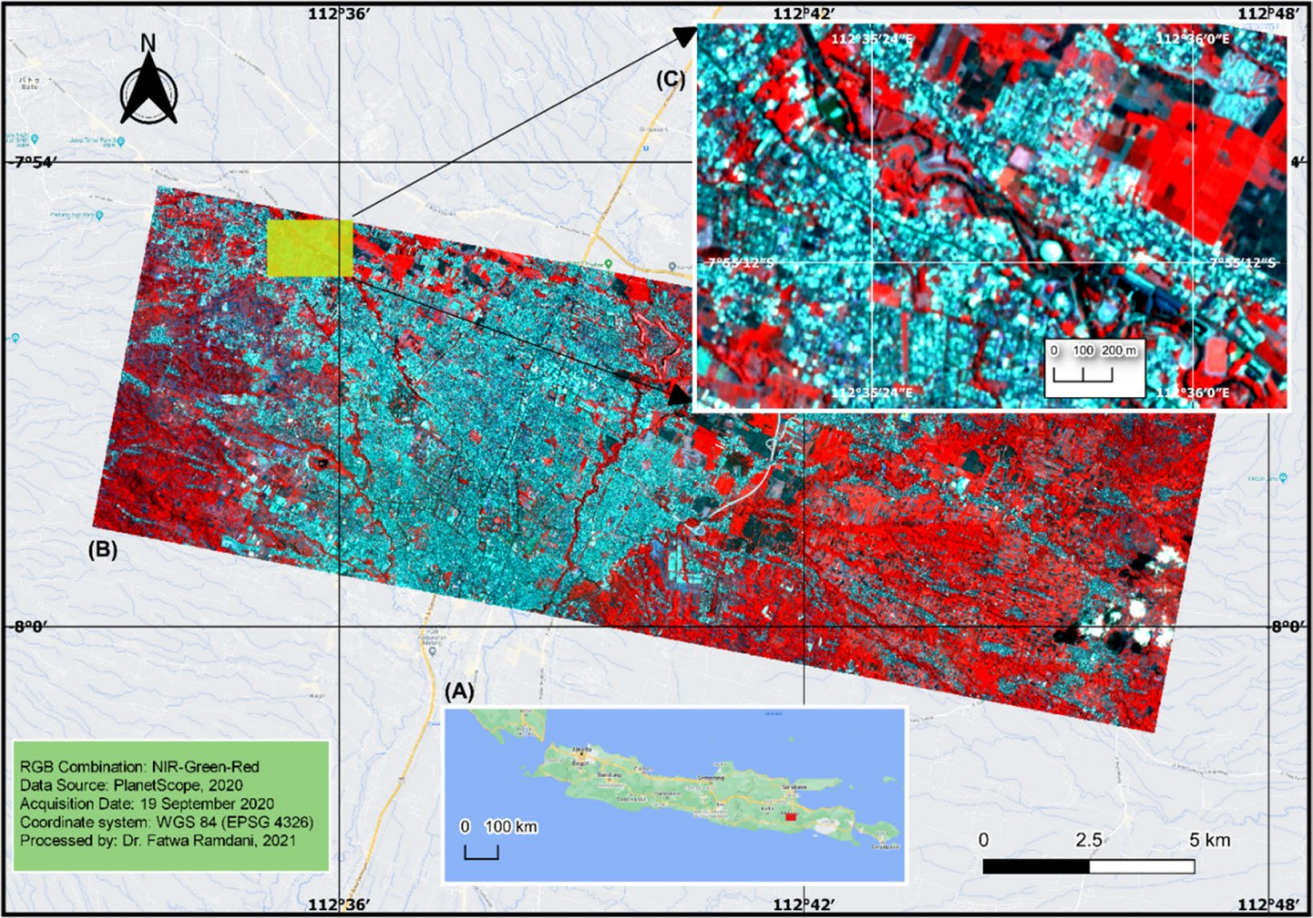
Study area located in East Java Province, Java Island (**A**), and Malang City (**B**). Satellite data and drone imagery were acquired from a suitable location northwest of Malang City, inside the Sengkaling Water Park (**C**) This figure was created using QGIS Desktop version 3.18.1, available at https://download.qgis.org/downloads/.

The Brantas River flows through the city of Malang. The study area is in East Java Province, northwest of Malang City, and is located within the Sengkaling Water Park (Fig. 1). This is a suitable location because it comprises an open space that is surrounded by a dense population. This study site is, therefore, suitable for monitoring and mapping large amounts of macroplastic waste.

### Methodology

PlanetScope data were obtained from the official page (https://www.planet.com/explorer/). The PlanetScope dataset was only used to perform overviewing and produce map of the study area as shown in Fig. 1. The unmanned aerial systems used was the DJI Mavic Pro. Very low altitude drone flights were planned with Pix4D application. The orthomosaic dataset was generated using Agisoft Metashape software, and QGIS was used to clip by region of interest (ROI), the riparian zones along the Brantas River.

**Figure 2:**
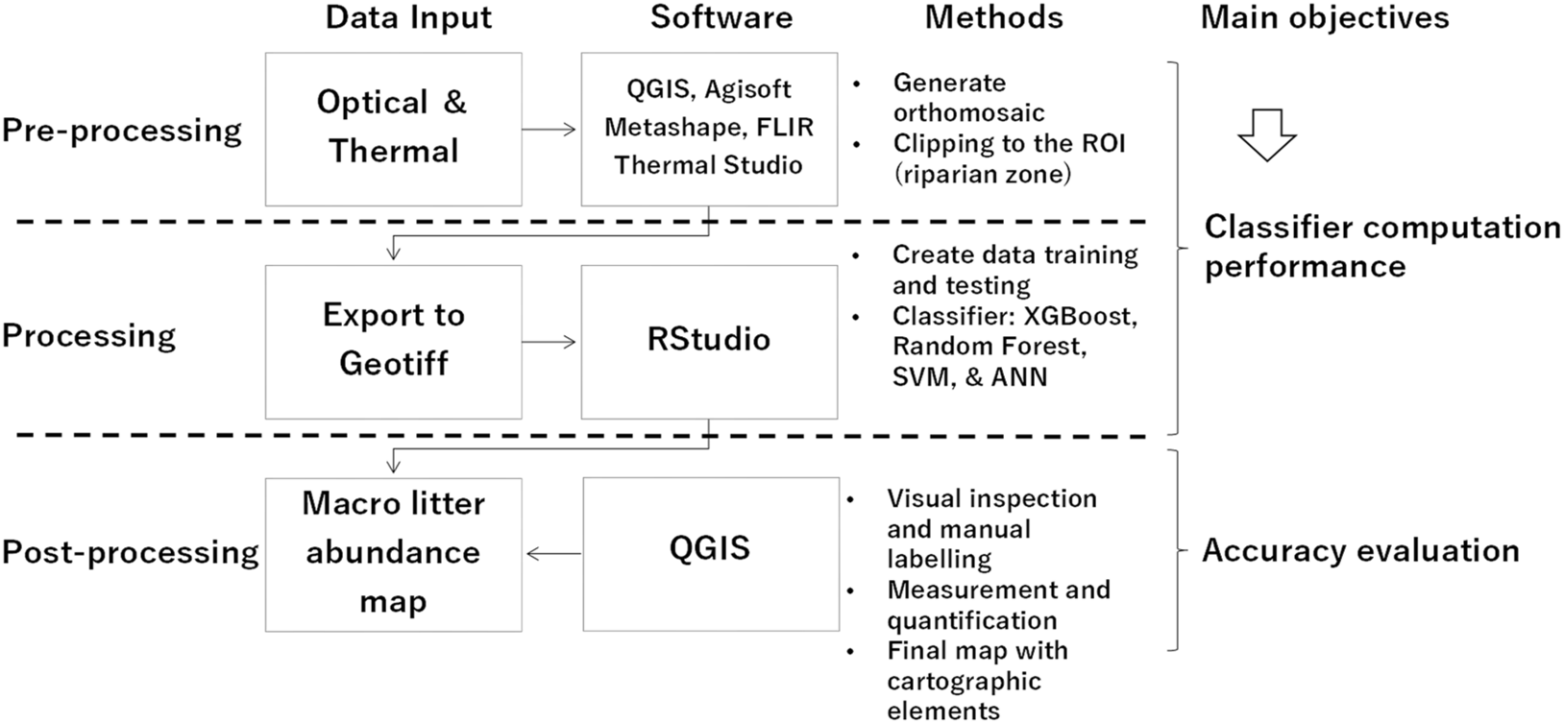
Proposed methodology for mapping macro litter in riparian zones using machine learning algorithms.

The data were exported to Geotiff, which was ready to be processed using RStudio with the four different machine learning methods (XGBoost, SVM, RF, and ANN). Certain packages (libraries) such as xgboost, caret, nnet, randomforest, kernlab, and e1701 were required to process the machine learning methods (Fig. 2). The model training was conducted offline using personal computer.

Because the focus of the study was on mapping the abundance of macroplastic waste in the riparian zone, a classification scheme was carefully selected and defined considering the appropriate classes (Table 1). The modified Pareto principle (CSU, 2020) was used to separate the training data (70%) and testing data (30%).

**Table 1:** Classification scheme and training sample size used in the study.

Class ID	Class names	Description	Number of samples
1	Water body	River surface water	10
2	Stone	All types of stones and boulders in the river	10
3	Vegetation	Vegetation above the surface water	10
4	Plastic	Macro litter abundance on the surface/submerged (5–10 cm)	10
5	Branch	Non-anthropogenic (vegetation) debris	10

### Data acquisition

The optical and thermal data were captured using a mobile device mounted on the UAS. The Forward Looking Infrared (FLIR) thermal sensor was a FLIR ONE PRO Micro USB Thermal Camera, with an infrared sensor size of 160 × 120 mm. The resulting pixel resolution was 19, 200 pixels with a thermal sensitivity of up to 0.07 ℃. The temperature range that can be recorded is between − 20 and 400 ℃.

**Figure 3:**
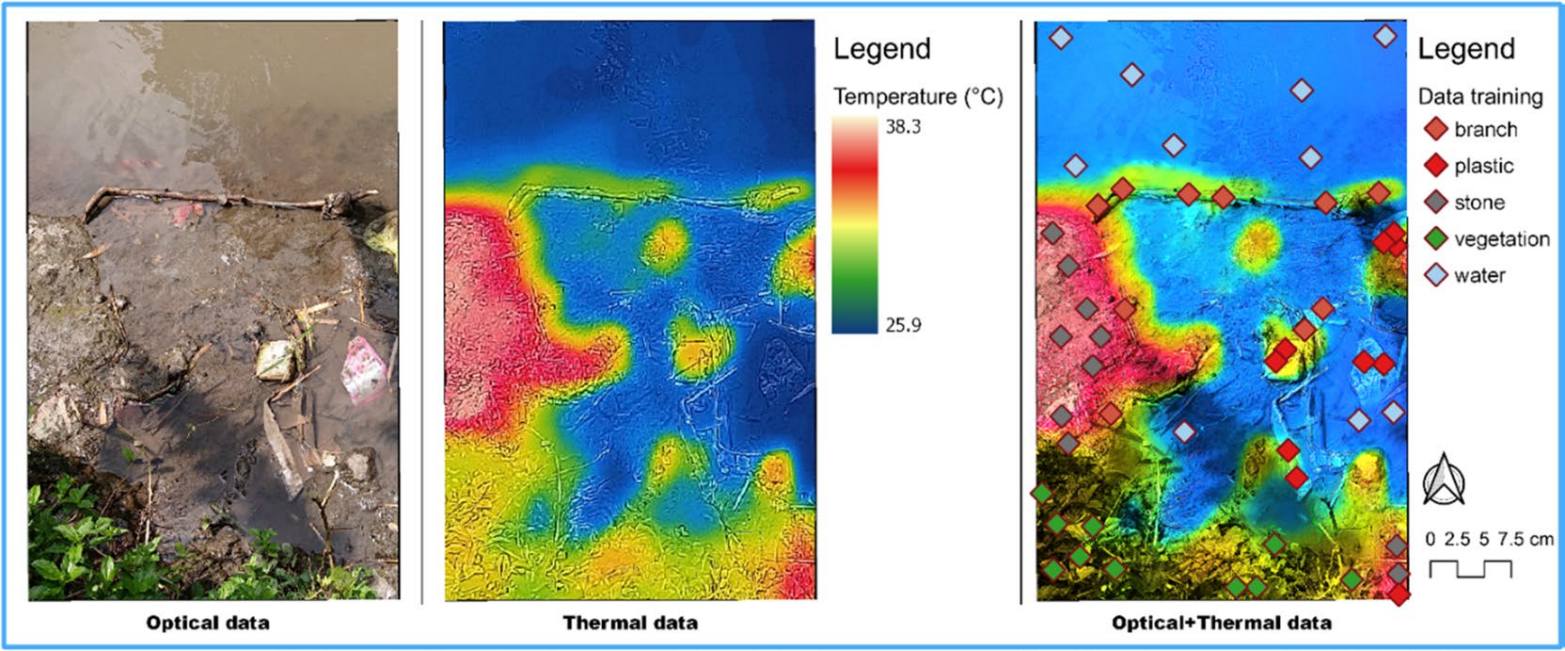
Clipped orthomosaic of optical and thermal sensor data, and a combination of both data and the distribution of the training data used around the riparian zone. This figure was created using QGIS Desktop version 3.18.1, available at https://download.qgis.org/downloads/.

The optical sensor had a focus of 15 cm to infinity, with a frame rate of 8.7 Hz and Field of View (FOV): Horizontal (HFOV) of approximately 50° ± 1°, Vertical (VFOV) of approximately 43° ± 1. The optical and thermal sensor data, and a combination of the two, as well as the point distribution used as training data, are shown in Fig. 3. To extract the temperature information for each recorded object, thermal data from the FLIR sensor were then further processed using FLIR Thermal Studio software. The submerged macro litter is at a depth of between 5 and 10 cm below the water surface.

### Accuracy assessment

**Figure 4:**
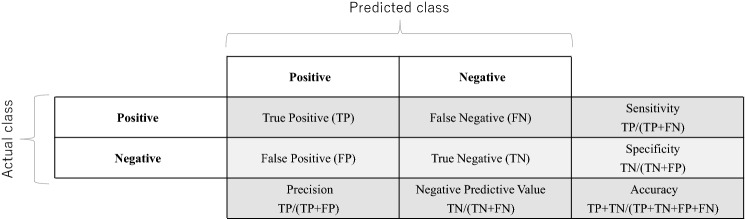
Confusion matrix.

An accuracy and performance assessment was performed using the confusion matrix and Cohen’s Kappa. The confusion matrix plots the number of correct predictions against the number of incorrect predictions. For the binary classifier, this means the number of true negatives and true positives (correct predictions) versus the number of false negatives and false positives (false predictions) (Fig. 4).

Cohen's Kappa is a statistical measure of the reliability of categorical variables. It is defined using Eq. (4):

4 $$k = \frac{{p_{o} + p_{e} }}{{1 - p_{e} }},$$

where *po* is the observed relative agreement among raters (identical to accuracy) and *pe* is the hypothetical probability of chance agreement, using the observed data to calculate the probability of each observer randomly viewing each category. If the raters are at the perfect level, then the value of *k* = 1.

Cohen’s Kappa can then be interpreted (Table 2).

**Table 2:** Cohen’s Kappa interpretation.

Values	Interpretation
< 0	There is no relationship
0.01–0.20	Low
0.21–0.40	Enough
0.41–0.60	Moderate
0.61–0.80	High
0.81–1.00	Perfect

## Results and discussion

### Fitting models

The training data were used to perform cross-validation and grid search for model tuning. Once the optimal/best parameters were found, the final model was matched for the entire training dataset using the findings. It was then determined how the final model behaved on the test dataset.

**Figure 5:**
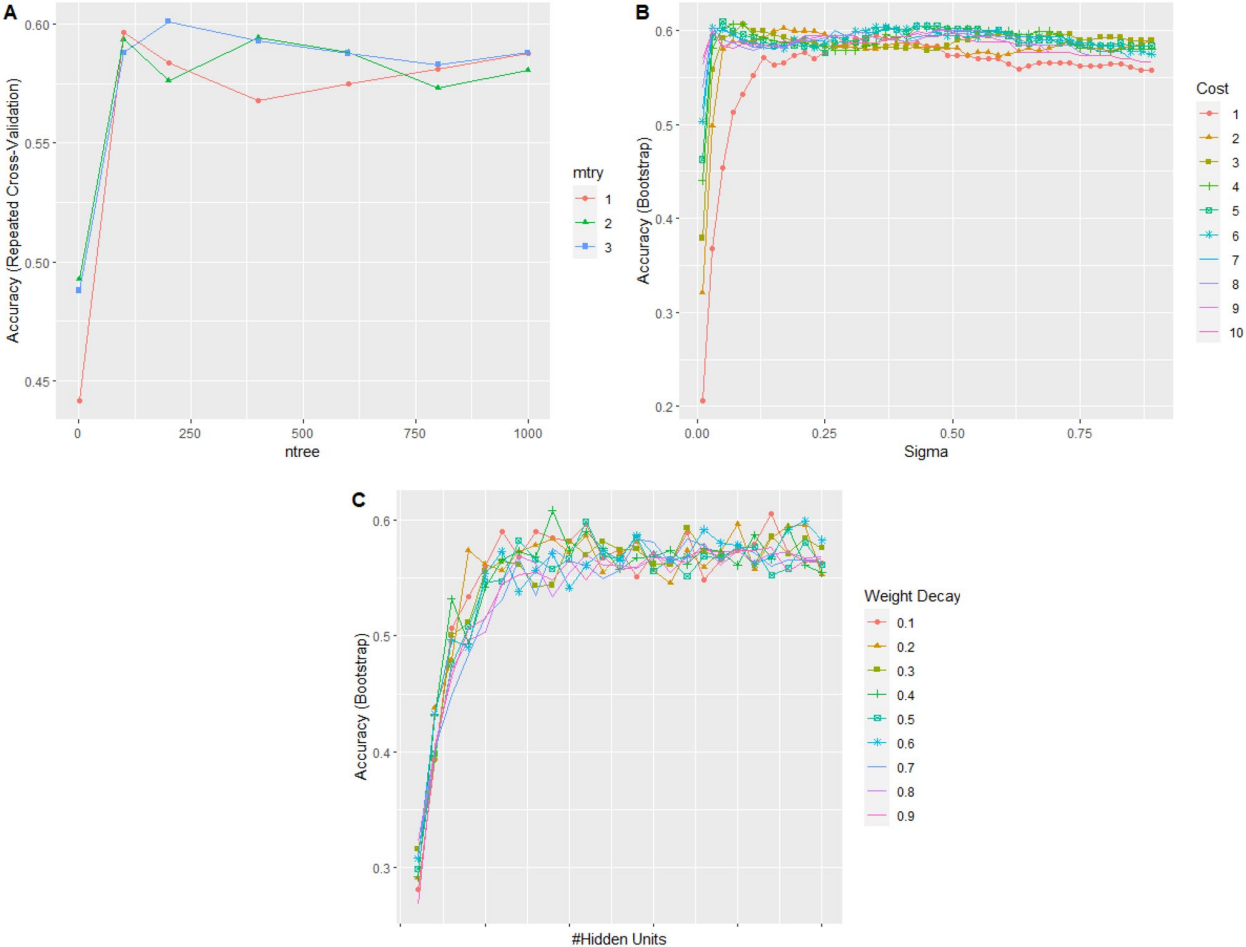
Relationship between parameters (RF (**A**), SVM (**B**), and ANN (**C**) algorithms) and accuracy.

The fitting models used in this study were set manually. Manual tuning involves selecting some desired value for setting the model parameters, and a fine-grained control over the tuning parameters can be obtained. Manual tuning was performed via the tuneGrid argument, as follows:

- mtry (number of randomly selected predictors) and ntree (number of branches that will increase after each split) parameters for the RF algorithm. In this study, a value range of 1–3 for the mtry parameter, according to RGB pixels was used, and the ntree used was 1–1000. The final values used for the best model were three for mtry and 200 for ntree.
- For cost and loss function (sigma) parameters (to control the nonlinearity of the hyperplane and the effect of each supporting vector) for the SVM algorithm, values in the range of 1–10 and 0.1–0.9 were used for the cost and sigma parameters, respectively, and the final values used to obtain the best accuracy were sigma = 0.05 and C = 5.
- For the size and decay parameters (number of neurons in the hidden layer and regularisation parameters to avoid overfitting conditions) for the ANN algorithm, the size value used was 1–25 and the decay value used was 0.1–0.9. The final values used to obtain the best model were: size = 9 and decay = 0.4. Figure 5 shows the results of manual tuning using the parameters defined during training for the RF, SVM, and ANN algorithms.

For the XGBoost algorithm, model tuning was performed as follows: The eta parameter was set to 0.1, max_depth was assigned a value of 6, subsample was assigned a value of 0.7, and colsample_bytree was assigned a value of 1.

**Figure 6:**
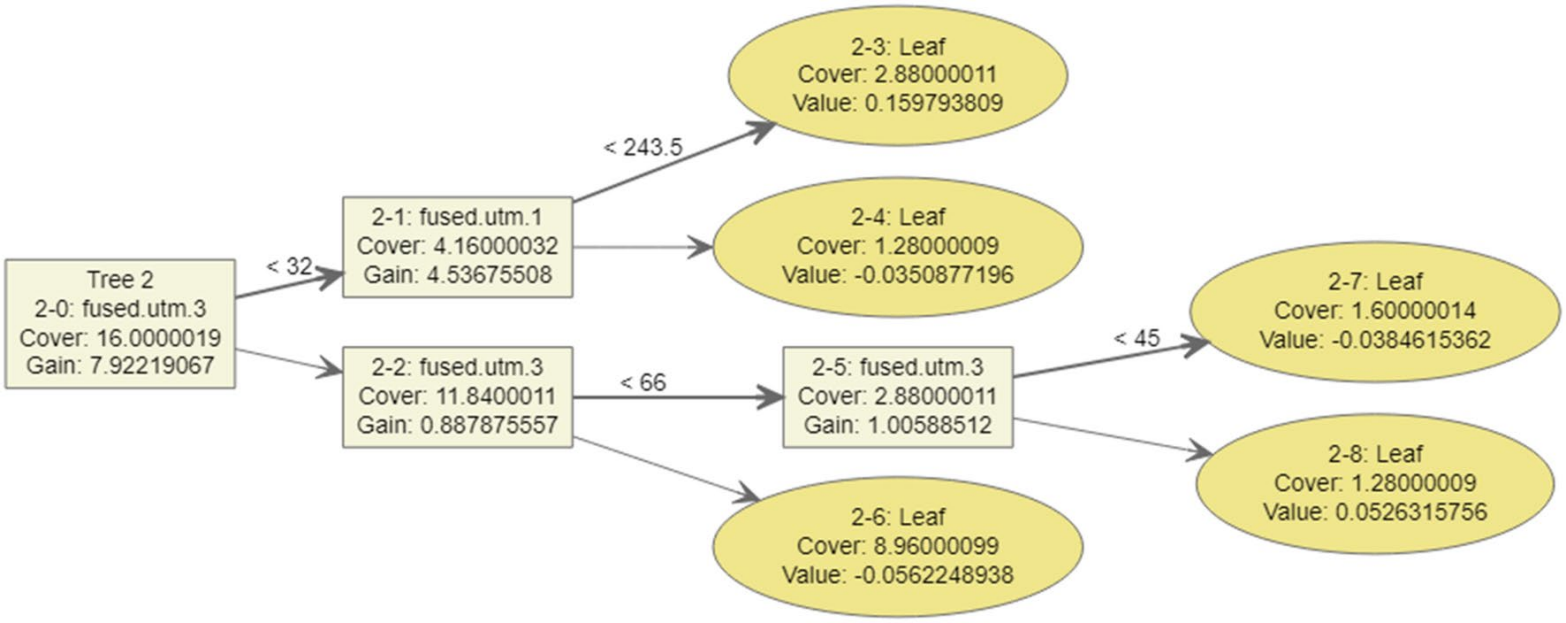
Sample of one of the result trees generated by XGBoost according to the specified parameters.

**Figure 7:**
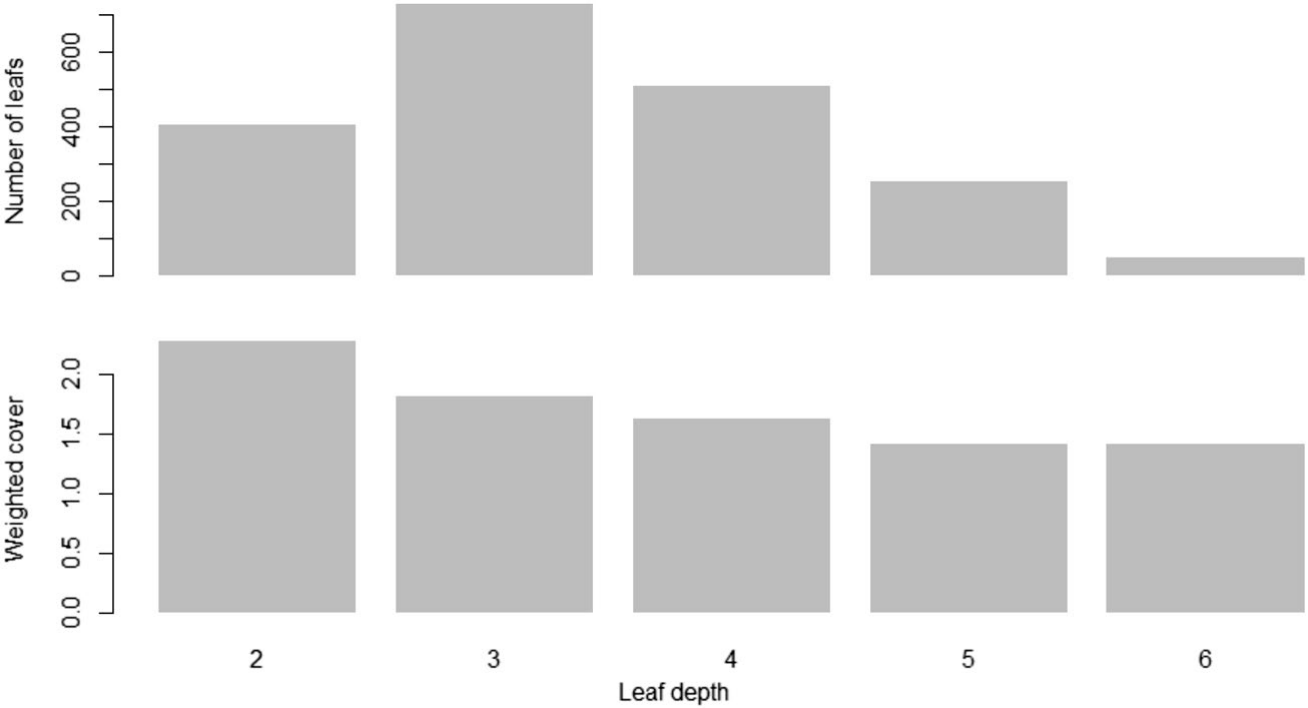
Complexity of the model generated by the XGBoost algorithm using predefined parameters.

To prevent overfitting, it was necessary to determine the step size reduction used in the weight updates. After each boosting step, new feature weights were immediately obtained, and eta reduced the feature weights to make the updating process more conservative. The step-size reduction values ranged from 0 to 1. A low eta value makes the prediction model easily trapped in overfitting conditions. The max_depth parameter was the maximum depth value of the tree, and it can vary from 1 to infinity. The subsample parameter was the ratio of the training dataset. A value of 0.5 means XGBoost will randomly collect half of the training dataset to create a tree, which will prevent overfitting conditions. The subsample parameter values ranged from 0 to 1. The colsample_bytree parameter was the subsample ratio of the columns when constructing each tree, and the values ranged from 0 to 1. Figure 6 illustrates one of the decision trees generated by XGBoost, and Fig. 7 presents a bar graph of the resulting model using predefined parameters.

### Pansharpening (optical and thermal data merge) and pixel values extraction

The process of pansharpening or combining optical and thermal data was crucial for this research. The steps must provide a false coordinate system for both the optical and thermal data.

The false coordinate assignment process used QGIS software with the Georeferencer feature. A georeferenced map-to-image approach was taken using Google Maps. Coordinates were determined by providing four main points in the upper right, upper left, lower right, and lower left corners of the optical and thermal images. In the transformation parameter, a linear transformation was used with the resampling nearest neighbour method.

Using these results, the two images data could then be combined using the pansharpening method utilising the cubic resampling algorithm. The coordinates played an important role in the data merge process. According to these results, the optical and thermal data given by the false coordinates did not overlap with each other precisely. There was a gap of several millimetres, which resulted in an imperfect data merge result. This had an impact on the classification process and results.

The classification process took point data with pixel values from each channel/band. Point data were obtained by randomly generating 10 points for each class, 50 points in total. The point-sampling tool from QGIS was used for the extraction process. These data were then used as training and test data after being divided using the modified Pareto principle.

Because of imperfect pansharpening, the extracted pixel values were not completely accurate, and consequently, the classification results were not completely accurate. This also occurred because the optical and thermal sensors were from different devices.

### Performance comparison

**Figure 8:**
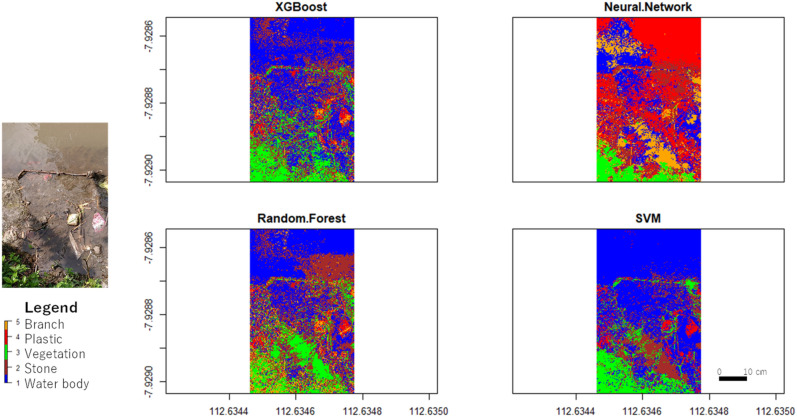
Optical data classification result.

**Figure 9:**
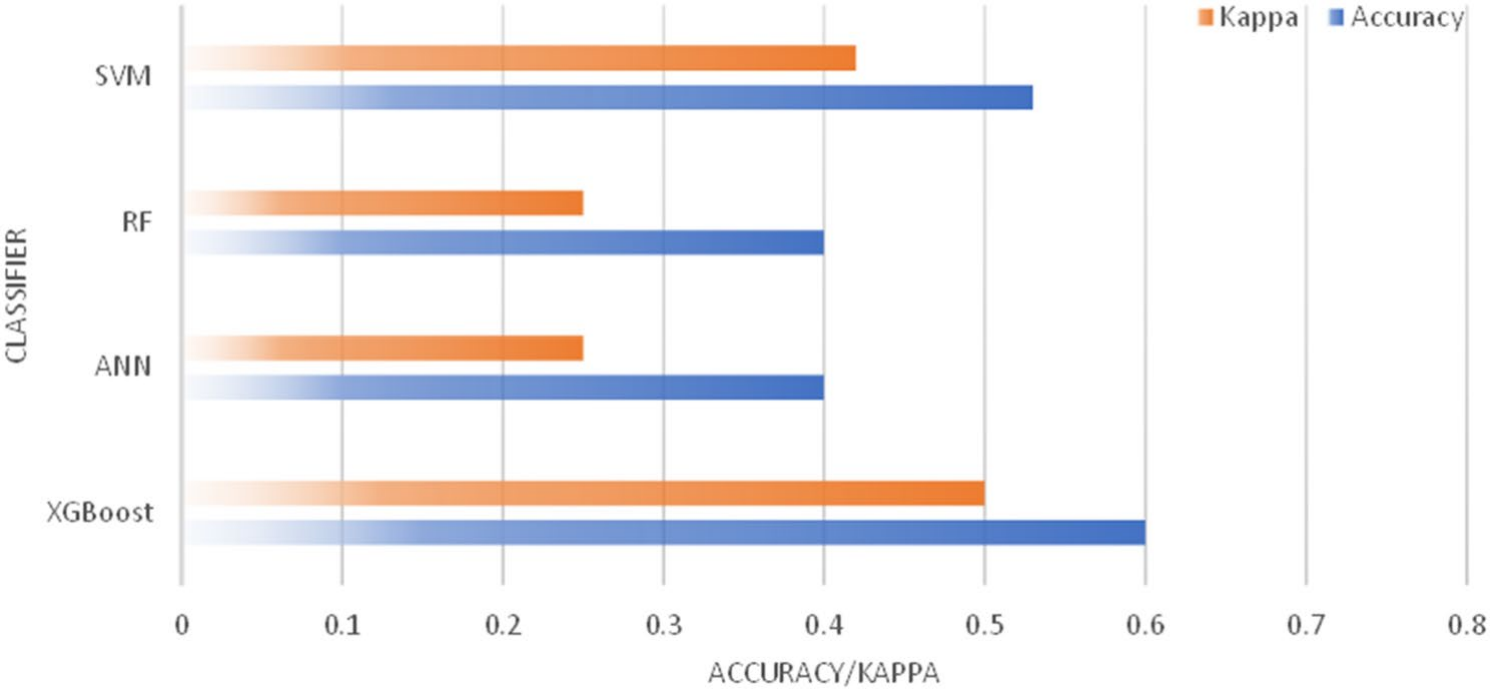
Accuracy and Cohen’s Kappa of XGBoost, Neural Network, Random Forest, and SVM using optical data.

The performance of each algorithm was compared in terms of accuracy and Cohen’s Kappa. The classification results of XGBoost, Neural Network, Random Forest, and SVM are shown in Fig. 8. The ANN and RF algorithms yielded low results, with an accuracy of 40% and Cohen’s Kappa of 0.25 (low). The SVM algorithm obtained an accuracy of 53% and Cohen’s Kappa of 0.42 (moderate). The XGBoost algorithm obtained an accuracy of 60% and Cohen’s Kappa of 0.5 (moderate). Figure 9 presents the accuracy and Cohen’s Kappa for each algorithm using the optical data input in graphics.

**Figure 10:**
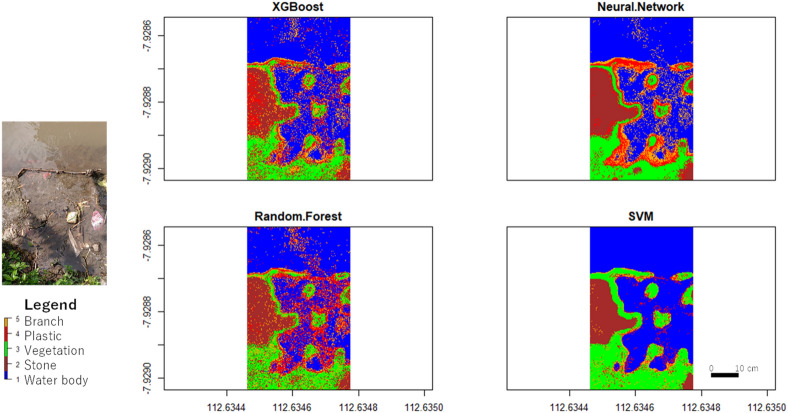
FLIR thermal data classification results.

**Figure 11:**
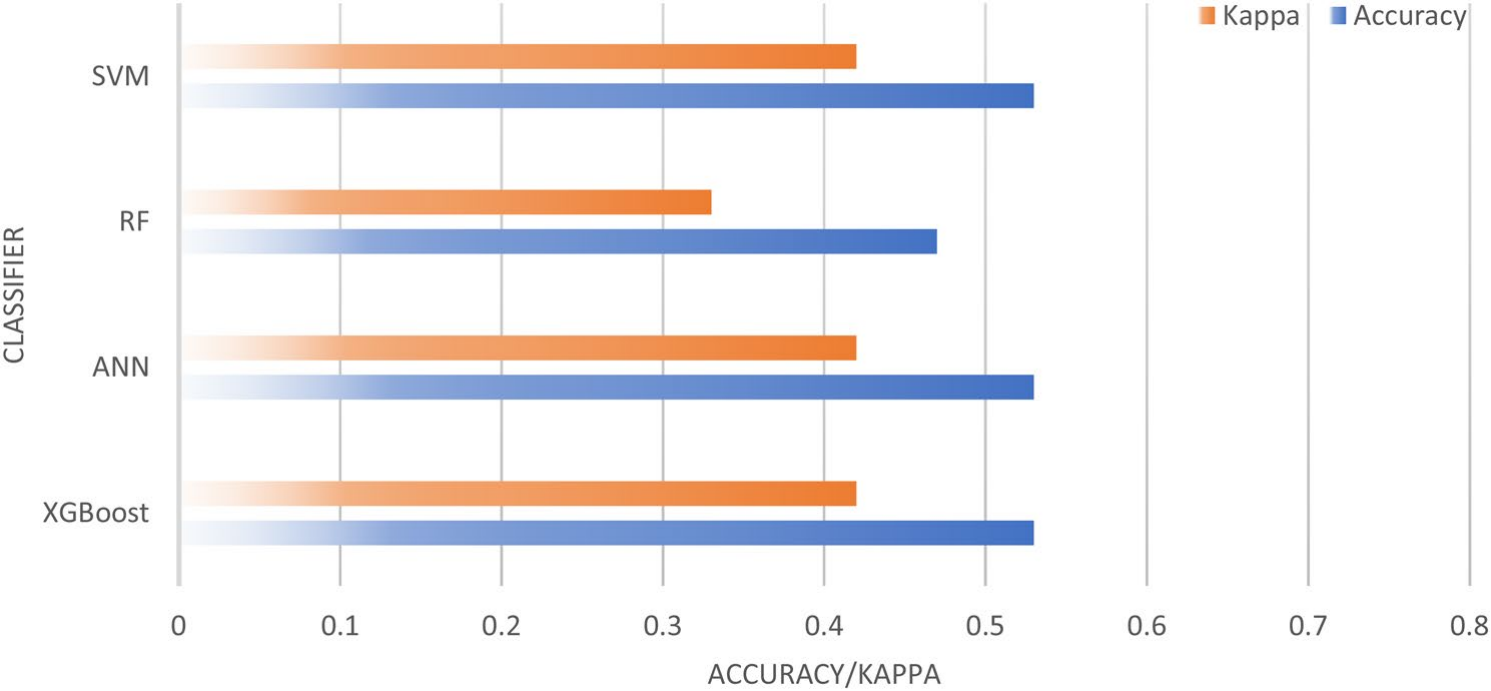
Accuracy and Cohen’s Kappa of XGBoost, Neural Network, Random Forest, and SVM using FLIR thermal data.

The classification results using the FLIR thermal data are shown in Fig. 10. XGBoost, ANN, and SVM yielded the same results, with an accuracy of 53% and Cohen’s Kappa of 0.42 (moderate). The RF algorithm obtained lower results, with an accuracy of 47% and Cohen’s Kappa of 0.33 (moderate). Figure 11 presents the accuracy and Cohen’s Kappa for each algorithm using the optical data.

**Figure 12:**
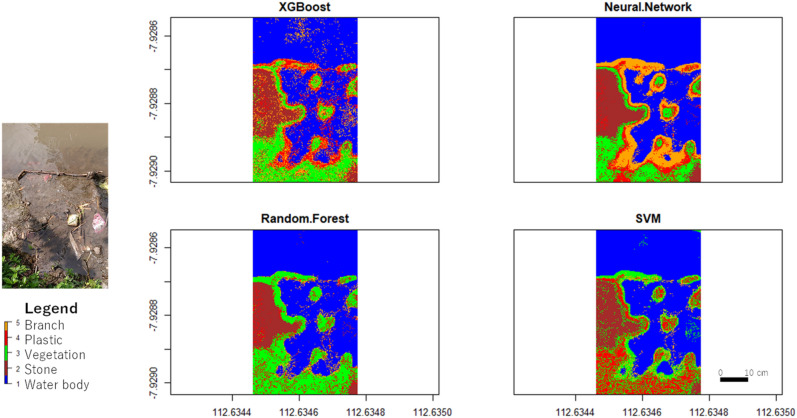
Results of data fusion classification (optical and thermal).

**Figure 13:**
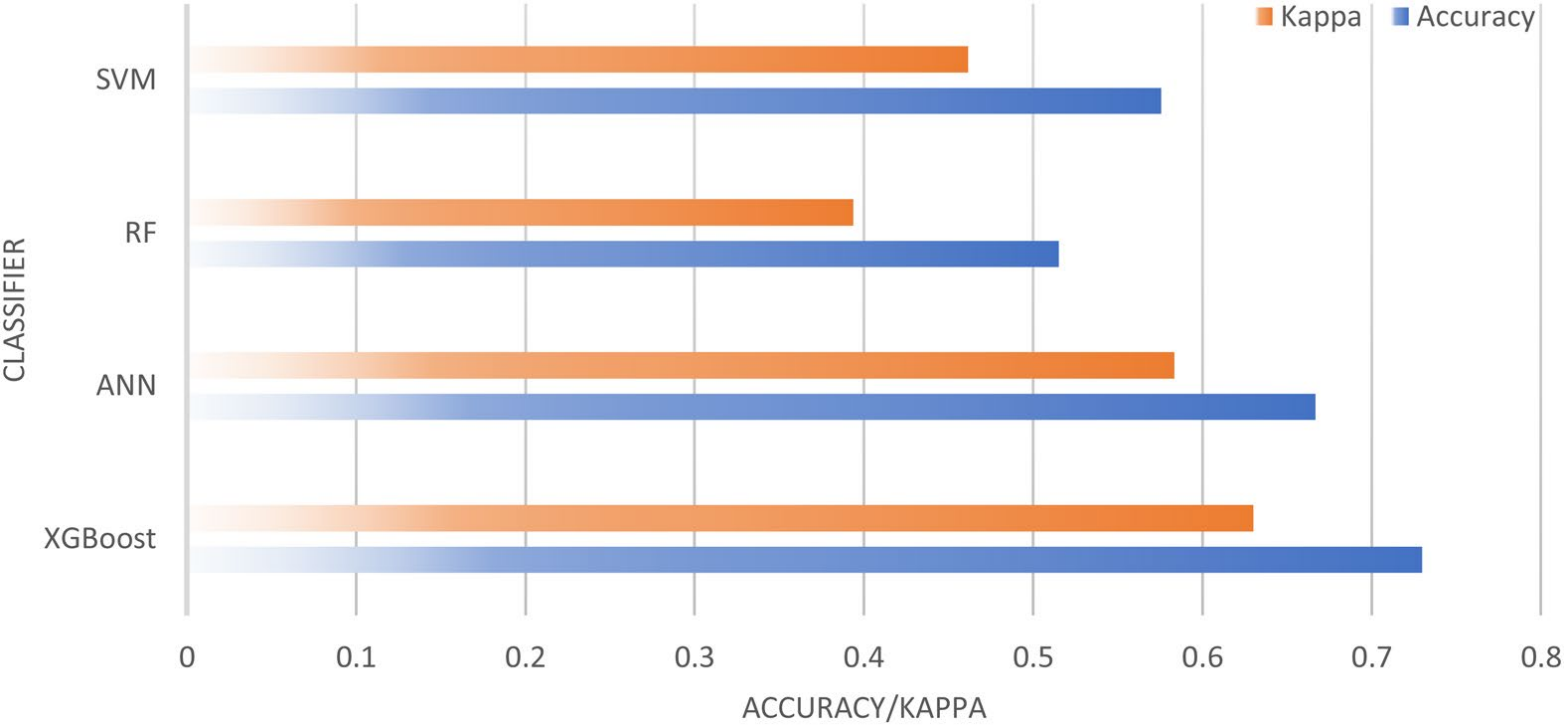
Accuracy and Cohen’s Kappa value of each algorithm using the FLIR optical and thermal combined data input.

Figure 12 presents the classification results obtained using the combined optical and thermal FLIR data, while Fig. 13 presents the accuracy and Cohen’s Kappa value for each algorithm using FLIR's combined optical and thermal input data in a graphical format The RF algorithm obtained an accuracy of 51% and Cohen’s Kappa of 0.39 (moderate). The XGBoost, ANN, and SVM algorithms obtained accuracies of 73%, 66%, and 57%, respectively. The Cohen’s Kappa value obtained by XGBoost was 0.63 (high), by ANN was 0.58 (moderate), and by SVM was 0.46 (moderate). The achieved accuracy is sufficient for practical uses^25^.

### Time performance comparison

**Figure 14:**
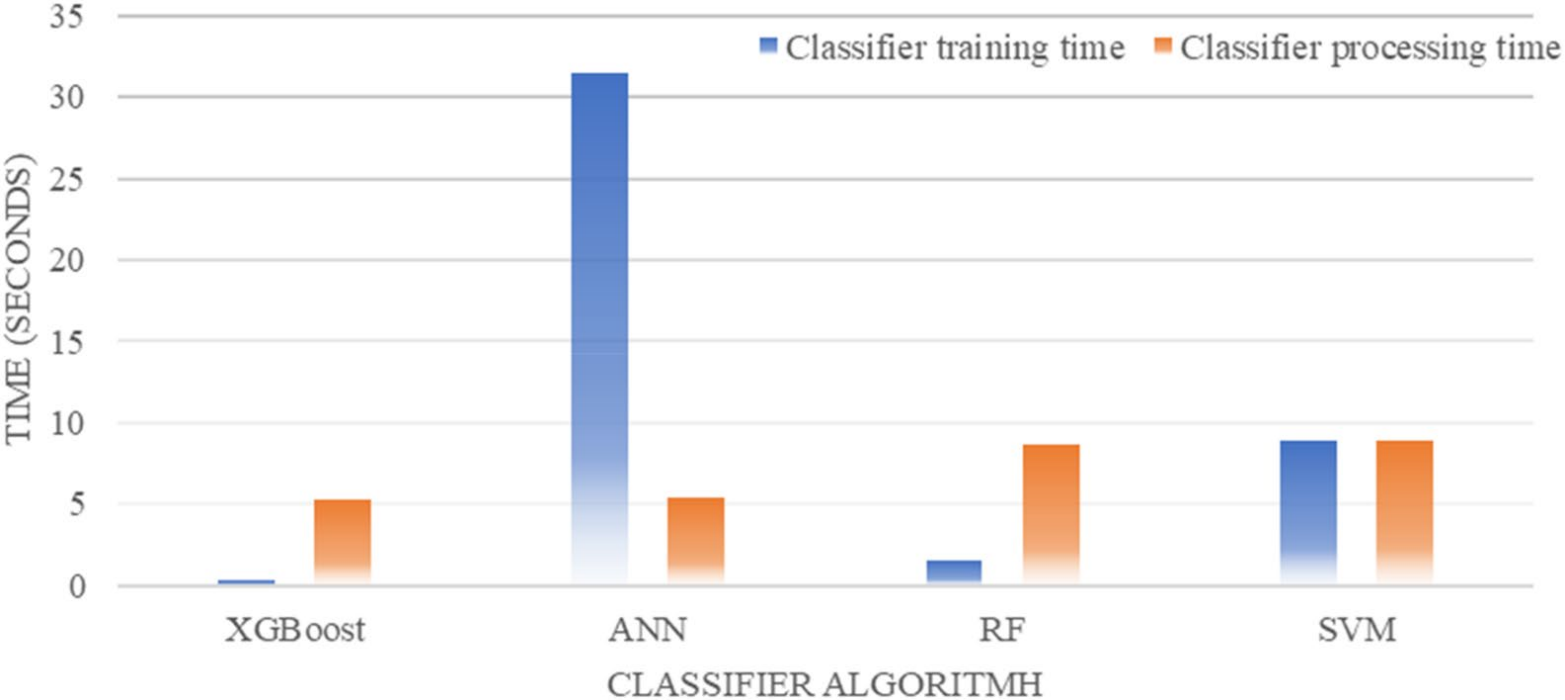
Time performance of each algorithm in the training and classification process.

The time performance for each process was performed among algorithms using the *profvis* package in RStudio. Figure 14 shows the average duration required for each algorithm to perform the training and classification processes.

The ANN algorithm had the most extended training duration, followed by SVM and RF. XGBoost had the most rapid training duration. The SVM and RF algorithms required a longer duration for the classification process than the ANN and XGBoost algorithms. XGBoost had the highest time performance in all stages, and the ANN algorithm was the least efficient.

### Plastic waste above and below the water surface

Plastic waste sources in the riparian zone of rivers vary, for example, single-use plastic packaging, shampoo, or soap wrappers. This plastic waste will not be decomposed and will continue to be in the riparian zone or be carried by water currents downstream.

**Figure 15:**
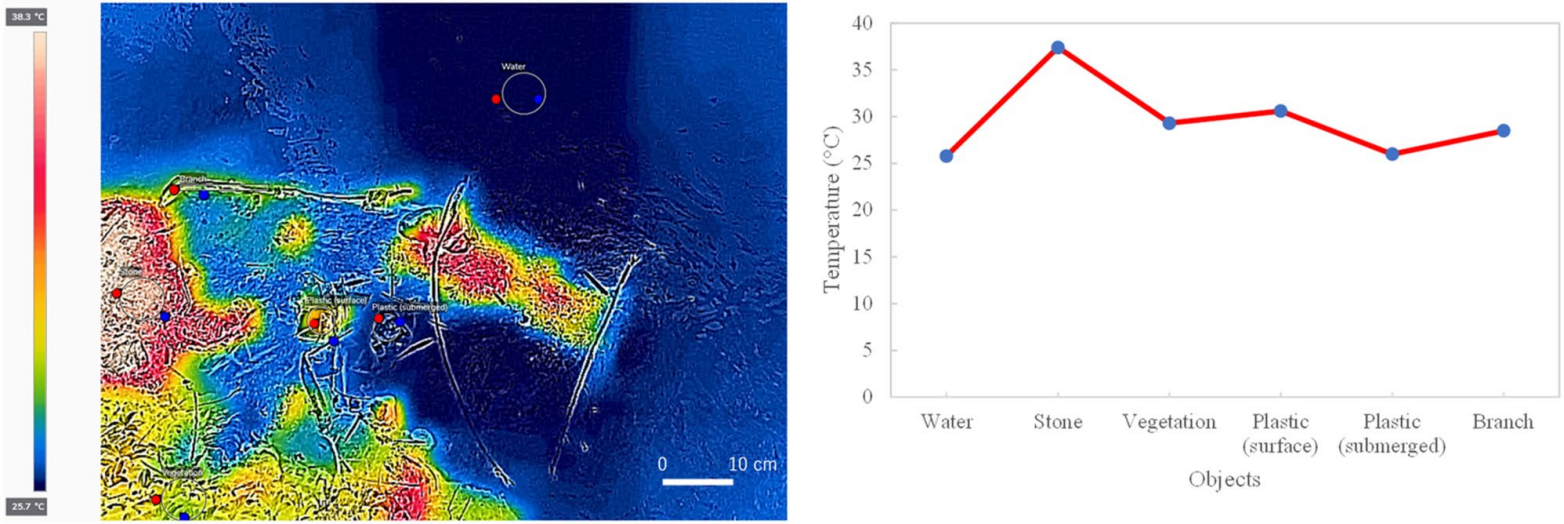
Temperature difference of each object. This figure was created using FLIR Thermal studio version 1.7.24, available at https://www.flir.com/products/flir-thermal-studio-suite/.

Plastic waste recorded by optical and thermal sensors is above and below the water surface, providing challenges for accurate classification results. Plastic waste below the water surface has a lower thermal value than that above the water surface. Often, the thermal value of plastic above the water surface is similar to that of other objects, such as vegetation and trunks/branches. The thermal value of plastic waste below the water surface is the same as the thermal value of the water (Fig. 15).

**Figure 16:**
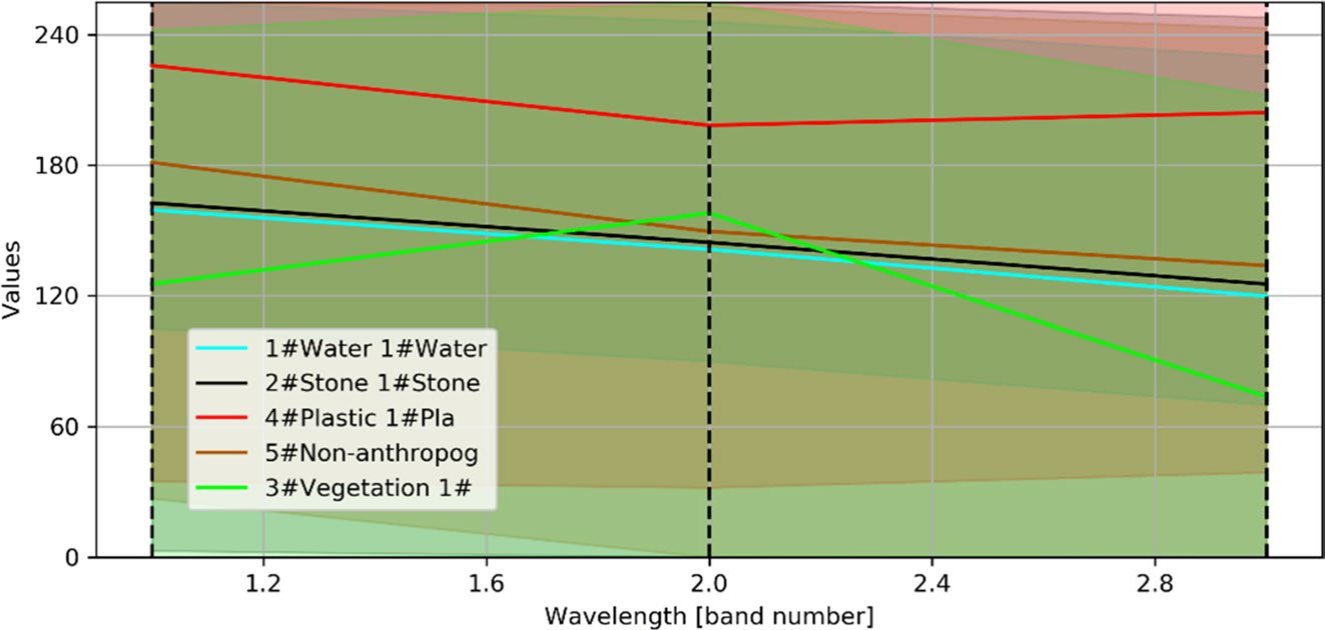
Spectral values difference of each object.

The optical sensor provides a better pixel value in distinguishing plastic waste from other objects, regardless of whether the garbage is above, or below, the water surface. The spectral value of plastic has the highest range of values, and vegetation has the lowest range of values compared to other objects (Fig. 16).

**Figure 17:**
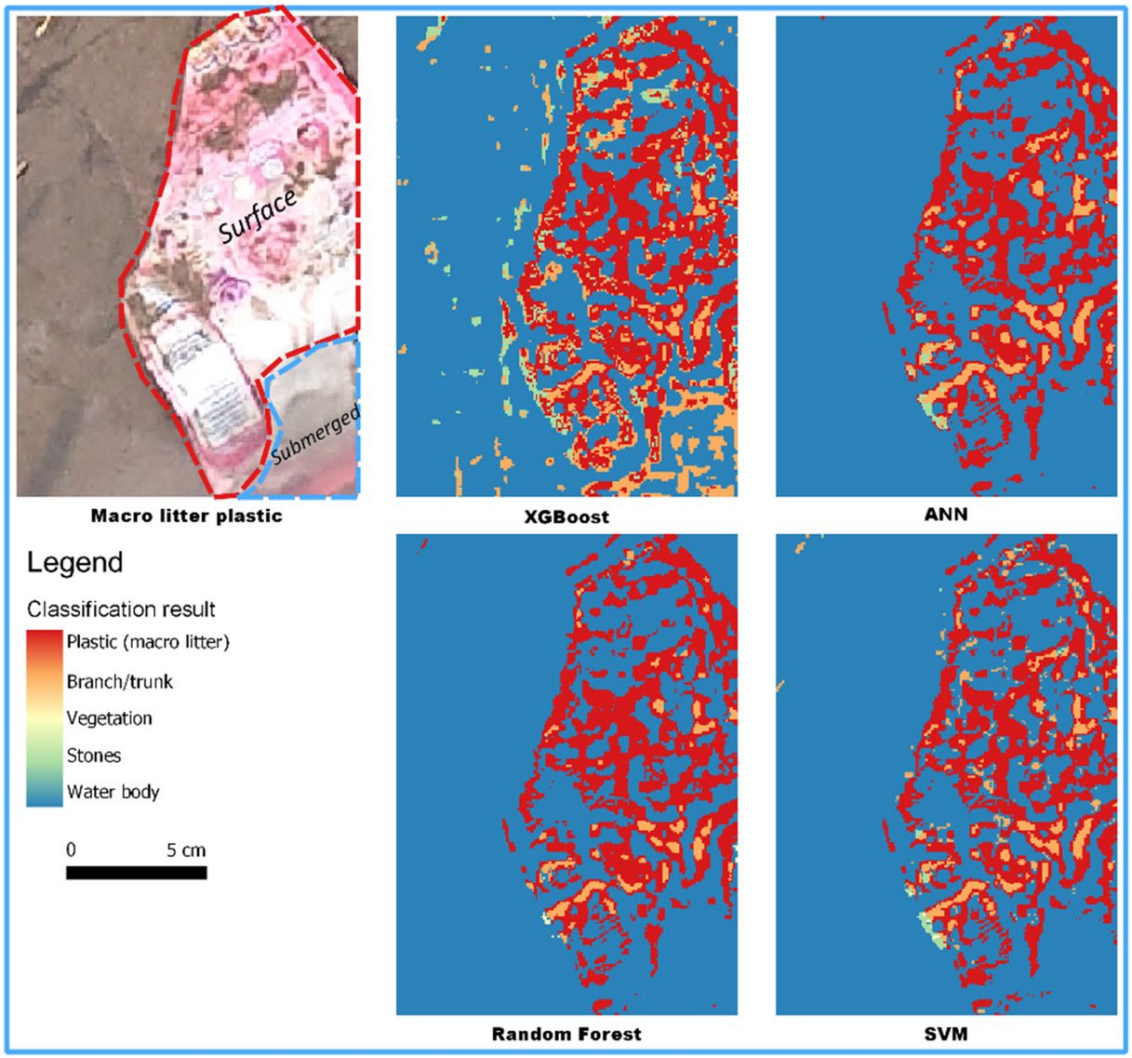
Comparison of plastic waste classification with four classification algorithms using combined optical and thermal data. The submerged macro litter plastic is at a depth of 5 cm. This figure was created using QGIS Desktop version 3.18.1, available at https://download.qgis.org/downloads/.

The combination of optical and thermal data yielded moderate results. The increase in accuracy and Cohen’s Kappa was not significant, despite the combination of optical and thermal data that was used in the classification process. The classification using the thermal data alone had 40–50% accuracy, and the optical data alone had 50–60% accuracy. The accuracy increased to 60–70% when using the merged data. Figure 17 compares the plastic waste classification results from the four classification algorithms using a combination of optical and thermal data. The plastic waste is above and below the surface. Plastic waste below the water surface is visible only in shallow and clear water, but not in deep and murky water. The classification results are unsatisfactory.

## Conclusion

This study demonstrates that optical, thermal, and a combination of both sensors can be used to map plastic waste in riparian zones. The combination uses a pansharpening technique with the cubic resampling method. The mapping uses supervised XGBoost, ANN, RF, and SVM classification algorithms, where the ratio of training data to test data is 70:30. The data collected were classified into five classes, namely, waterbody, stone, vegetation, plastics, and branch.

The results indicate that using only one data source as the input results in low accuracy and a low Cohen’s Kappa value. Using only FLIR thermal data, all classification algorithms can only obtain accuracy results of 47–53% and a Cohen’s Kappa value of 0.33–0.42 (moderate). A slight increase in accuracy is achieved when using optical data alone at 53–60% and a Cohen’s Kappa value of 0.42–0.50 (moderate). The accuracies of all algorithms increase when the combination of the two data is used at 60–73% and a Cohen’s Kappa value of 0.63–0.73 (high). The classification accuracy depends on the application, the accuracy of more than 70% is sufficient for practical uses since it is only for macro litter classification. For further observation, the accuracy can be improved with 80:20 data training and testing strategy.

The durations of the training and classification processes were evaluated. The ANN algorithm has the longest duration for the training process. For the classification process, all algorithms have the same relative duration. It can be concluded that XGBoost is the most efficient classification algorithm compared to the other three classification algorithms.

The research challenges are related to the dynamics of the study area. First, the location of plastic waste above, and below, the water surface influences the classification results. The imperfect false coordinates leading to the inaccurate overlap of the optical and thermal data also influence the results.

Future research is recommended. Similar studies should include thermal value correction, which was not performed in this study. Other supervised classification algorithms can be compared to evaluate their performances in mapping plastic waste. Methods for improving data georeferencing are suggested.
